# Sustained Beta-Cell Dysfunction but Normalized Islet Mass in Aged Thrombospondin-1 Deficient Mice

**DOI:** 10.1371/journal.pone.0047451

**Published:** 2012-10-19

**Authors:** Carl Johan Drott, Johan Olerud, Hanna Emanuelsson, Gustav Christoffersson, Per-Ola Carlsson

**Affiliations:** 1 Department of Medical Cell Biology, Uppsala University, Uppsala, Sweden; 2 Department of Immunology, Genetics and Pathology, Uppsala University, Uppsala, Sweden; 3 Department of Medical Sciences, Uppsala University, Uppsala, Sweden; University of Bremen, Germany

## Abstract

Pancreatic islet endothelial cells have in recent years been shown to support beta-cell mass and function by paracrine interactions. Recently, we identified an islets endothelial-specific glycoprotein, thrombospondin-1 (TSP-1), that showed to be of importance for islet angiogenesis and beta-cell function in young mice. The present study aimed to investigate long-term consequences for islet morphology and beta-cell function of TSP-1 deficiency. Islet and beta-cell mass were observed increased at 10–12 weeks of age in TSP-1 deficient mice, but were normalized before 16 weeks of age when compared to wild-type controls. Islet vascularity was normal in 10–12 and 16-week-old TSP-1 deficient animals, whereas islets of one-year-old animals lacking TSP-1 were hypervascular. Beta-cell dysfunction in TSP-1 deficient animals was present at similar magnitudes between 10–12 and 52 weeks of age, as evaluated by glucose tolerance tests. The insulin secretion capacity *in vivo* of islets in one-year-old TSP-1 deficient animals was only ∼15% of that in wild-type animals. Using a transplantation model, we reconstituted TSP-1 in adult TSP-deficient islets. In contrast to neonatal TSP-1 deficient islets that we previously reported to regain function after TSP-1 reconstitution, adult islets failed to recover. We conclude that TSP-1 deficiency in islets causes changing vascular and endocrine morphological alterations postnatally, but is coupled to a chronic beta-cell dysfunction. The beta-cell dysfunction induced by TSP-1 deficiency is irreversible if not substituted early in life.

## Introduction

The pancreatic islets are among the most vascularized of all organs in the body with the capillary network having a glomerular-like angioarchitecture. This means that in the islet, each beta-cell is surrounded by at least one islet endothelial cell, and therefore these cells by necessity are exposed to each other’s products [1]. In fact, the high islet vascular density and the vascular phenotype, with a great number of fenestrations, is induced and maintained by secretion of vascular endothelial growth factor from the beta-cells [2], [3], [4]. We and others have shown that the endothelial cells signal back to the endocrine cells and contribute to maintenance [5], and during certain conditions even expansion of the adult beta-cell mass [6]. Islet endothelial cells also support beta-cell function and contribute to enhanced glucose-stimulated insulin release and diminished internal degradation of insulin in the cells [7]. Indeed, the fraction of most vascularized islets respond better to a glucose load than other islets [8]. Important endothelial cell products mediating beta-cell supporting effects are vascular basement membrane proteins, particularly laminins [5], [7], as well as hepatocyte growth factor [6]. Recently, we identified another factor, the glycoprotein thrombospondin-1 (TSP-1), that in islets is more or less exclusively produced by the endothelium, and of importance for islet morphology and beta-cell function [9]. TSP-1 is mainly known for its anti-angiogenic properties [10], but the absence of TSP-1 has also been reported to cause hypervascular and hyperplastic islets in neonatal TSP-1 deficient animals due to its ability to activate transforming growth factor beta-1 (TGFβ-1) [11]. These animals were recently found to be markedly glucose intolerant, despite having an increased beta-cell mass, and islets with decreased glucose-stimulated insulin release and capacity for (pro)insulin biosynthesis [9]. The present study aimed to investigate long-term consequences for islet morphology and beta-cell function in TSP-1 deficient mice by a follow-up investigation of animals up to one year of age.

## Materials and Methods

### Ethics Statement

The study was carried out in strict accordance with the recommendations in the Guide for Care and Use of Laboratory Animals of the National Institutes of Health. The protocol was approved by the animal ethics committee for Uppsala University (Permit number: C360/9). Surgery was performed under 2.2.2-tribromoethanol anesthesia, and all efforts were made to minimize suffering.

### Animals

TSP-1 deficient (−/−) animals were generated by homologous recombination in 129/Sv-derived ES cells implanted in C57BL/6 blastocysts. Offsprings, backcrossed (N9) to a C57BL/6 genetic background, were generously donated by Jack Lawler (Beth Israel Deaconess Medical Center and Harvard Medical School, Boston, MA, USA). Wild-type TSP-1 (+/+) or TSP-1 (−/−) deficient mice at 10–12 weeks, 16 weeks or one year of age were allocated to the different studies. All mice were housed in a room with a 12 h light/12 h dark cycle with free access to tap water and pelleted food throughout the course of the study.

### Islet Endocrine Morphology

Pancreata from 10–12-week-old, 16-week-old and one-year-old wild-type and TSP-1 deficient mice were carefully dissected free of fat and lymph nodes and weighed. They were then fixed in 10% (vol/vol) formaldehyde and embedded in paraffin. Sections (5 µm thick) of pancreata were prepared and stained with an antibody for insulin (ICN Biomedicals, Aurora, OH, USA) or for glucagon (Novo Nordisk, Bagsvaerd, Denmark) in order to determine the percentage of beta-cells and alpha-cells in the different samples [12]. The slides were counterstained with hematoxylin. For each animal, ten or more tissue sections from all parts of the pancreas were randomly chosen and evaluated. The fraction of the pancreas composed of the endocrine tissue was measured by a direct point-counting method [13], where the fraction of intersections of islets was determined and used for calculations of the endocrine mass compensating for differences in pancreatic weight between animals. Thereafter, also the number of islets per square mm was counted and the islets divided into three groups depending on their mean diameter in investigated sections, either big (<200 µm), medium (25–200 µm or small (<25 µm). The mean value for and the percentage of each group of islets were calculated for each animal. The alpha- and beta-cell fraction of the islets was estimated by using the same technique measuring the alpha-cell, beta-cell and the whole islet area, respectively, and from these values the fraction of islet tissue occupied by the different cell types was counted. All analyses were performed by using a computerized system for morphometry (ImageJ 1.2v; National Institutes of Health, ML, USA).

### Islet Vascular Density

Sections (5 µm thick) of paraffin-embedded pancreatic samples obtained from 10–12-week-old, 16-week-old or one-year-old wild-type and TSP-1 deficient mice (see above) were stained with the lectin Bandeiraea simplicifolia (BS-1; Sigma-Aldrich, Irvine, UK), as previously described [12], [14]. The slides were counterstained with hematoxylin. In each animal, 10 or more sections from all parts of the pancreas were evaluated after staining with BS-1. The number of stained blood vessels in the islets was quantified under a microscope (magnification, X400) by point-counting [13]. The area of the investigated islets was determined by using Image J (ImageJ 1.2v; National Institutes of Health). Vascular density, i.e. the number of stained blood vessels per measured islet area (mm^2^) was then calculated.

### Islet Blood Flow

Blood flow measurements in 10–12-week-old wild-type and TSP-1 deficient mice were performed as previously described [15]. Briefly, the animals were anesthetized with an intraperitoneal injection of 0.02 ml/g body weight of avertin [a 2.5% (vol/vol) solution of 10 g 97% 2.2.2-tribromoethanol (Sigma, St Louis, MO, USA) in 10 ml 2-methyl-2-butanol (Kemila, Stockholm, Sweden) and placed on an operating table maintained at body temperature. An injection of 200 IU heparin (5000 IU/ml; Leo, Malmo, Sweden) was given in the left jugular vein. Polyethylene catheters were inserted into the ascending aorta, via the right carotid artery, and into the right femoral artery. The former catheter was connected to a pressure transducer to monitor mean arterial blood pressure. Approximately 9×10^4^ non-radioactive black microspheres (E–Z Trac; IMT, Irvine, CA, USA) with a diameter of 10 µm were injected during 10 sec via the catheter with its tip in the ascending aorta. Starting 5 sec before the microsphere injection and continuing for a total of 60 sec, an arterial reference blood sample was collected by free flow from the catheter in the femoral artery at a rate of approximately 0.10 ml/min. The exact withdrawal rate in each experiment was confirmed by weighing the sample. The animals were killed and the whole pancreas and the adrenal glands were carefully dissected free from fat and lymph nodes, blotted, weighed and placed between object slides. Before placement between object slides, each pancreas was cut into 20–24 pieces. The slides were then placed at −20°C for at least 24 h. This enables the visualization of both islets and microspheres, as the exocrine parenchyma becomes transparent when viewed after thawing in a microscope equipped with darkfield illumination [16]. The percent islet volume was determined by point-counting [13], [15]. Approximately 20–24 different fields were counted in each mouse pancreas (corresponding to ≈2400 points). The total contents of microspheres in the exocrine and endocrine parts of the pancreas, and in the adrenal glands were then counted under a light microscope. The number of microspheres in the arterial reference sample was determined by transferring the blood to glass microfiber filters with a pore size of 0.2 µm (Whatman, London, UK) and counting the microspheres under a microscope. The blood flow values were calculated according to the formula Q_org_ = Q_ref_ x N_org_/N_ref_, where Q_org_ is organ blood flow (ml/min), Q_ref_ is withdrawal rate of reference sample (ml/min), N_org_ is number of microspheres present in the organ, and N_ref_ is number of microspheres present in the reference sample. The microsphere contents of the adrenal glands were used to confirm that the microspheres were adequately mixed in the arterial circulation. A difference less than 10% in numbers of microspheres between the right and left adrenal gland was taken to indicate sufficient mixing. When the islet blood flow was expressed per islet weight, to correct for differences in size of the islet organ, the latter was estimated by multiplying the pancreatic weight with the islet volume fraction of the whole pancreas in each animal.

### Glucose Tolerance Test

Non-fasted wild-type C57BL/6 and TSP-1 deficient mice were injected with D-glucose (2.5 g/kg body weight; 300 mg/ml; Fresenius Kabi, Uppsala, Sweden) into the tail vein. Just before glucose injection a blood sample was taken from the cut tip of the tail of the animals and analysed for plasma glucose concentration by glucose reagent strips (Freestyle lite, Abbott, Alameda, CA, USA). Plasma glucose concentrations were then also measured at 10, 30, 60 and 120 min after glucose administration. A retroorbital blood sample for serum insulin analysis was collected 10 min after glucose injection and analyzed with an enzyme linked immunosorbant assay (ELISA; Mercodia, Uppsala, Sweden).

### Islet Transplantation and Graft Perfusion

We have earlier shown that TSP-1 and thereby TGFβ-1 can be reconstituted in TSP-1 deficient neonatal islets by transplantation into wild-type TSP-1 (+/+) recipients [9]. In order to investigate whereas the functional defect observed in TSP-deficient islets of adult mice is reversible, islets derived from 10–12 week-old TSP-1 deficient mice were implanted into either wild-type or TSP-1 deficient mice. Likewise, wild-type islets were transplanted into either 10–12-week-old wild type or TSP-1 deficient mice. For this purpose, pancreatic islets were isolated by collagenase digestion [17], and maintained free-floating in groups of 150 islets at 37°C (air/CO_2_ 95∶5) for 5–7 days in 5 ml culture medium composed of RPMI 1640 medium supplemented with 2 mmol/l glutamine, 11 mmol/l glucose, 10% (vol/vol) fetal calf serum, 100 U/ml sodium benzylpenicillate, (Roche Diagnostics, Scandinavia, Bromma, Sweden) and 0.1 mg/ml streptomycin. For transplantation, groups of 150 wild-type or TSP-1 deficient islets were packed in a braking pipette and syngeneically implanted beneath the capsule of the left kidney in wild-type or TSP-1 deficient C57Bl/6 mice anesthetized with avertin (cf. above). One month post-transplantation glucose-stimulated insulin secretion of the islet grafts was evaluated [18]. The graft-bearing left kidney was removed together with a part of the aorta and inferior vena cava. The ureter and the renal vein were cut, while the aorta was cannulated and infused with a continuously gassed (O_2_:CO_2_ (95∶5) Krebs-Ringer bicarbonate buffer supplemented with 2.0% (wt/vol) each of BSA (fraction V; Miles Laboratories, Slough, U.K.) and dextran T70 (Pharmacia, Uppsala, Sweden). For different time period during the infusion, the medium contained either 2.8 or 16.7 mmol/l D-glucose. The medium was administered at a rate of 1 ml/min without recycling for 60 min with a perfusion pressure of ∼40 mmHg. The perfusion experiment started with a 15-min period using medium containing 2.8 mmol/l glucose, which was followed by 30 min using 16.7 mmol/l glucose. The perfusions were concluded by a 15-min perfusion with medium containing 2.8 mmol/l glucose. 1.0 ml samples were collected at 14, 15, 16, 17, 18, 19, 20, 22, 25, 30, 35,40, 45, 50, 55 and 60 min. Insulin concentrations of the effluent samples were measured by ELISA (Mercodia). The rate of insulin secretion was calculated by multiplying the insulin concentration in the sample by the flow rate.

### Statistical Analysis

All values are given as means ±SEM. Parametric data with only two groups were analysed using Student’s unpaired two-tailed t-test, whereas non-parametric values were compared using non-parametric Wilcoxon’s signed rank test. Multiple comparisons were performed using ANOVA and Bonferroni’s post-hoc test. For all comparisons, a P value of <0.05 was considered to be statistically significant. Sigmaplot® (SPSS science software, Erfart, Germany) was used for the statistical analyses.

## Results

### Islet Morphology

**Figure 1:**
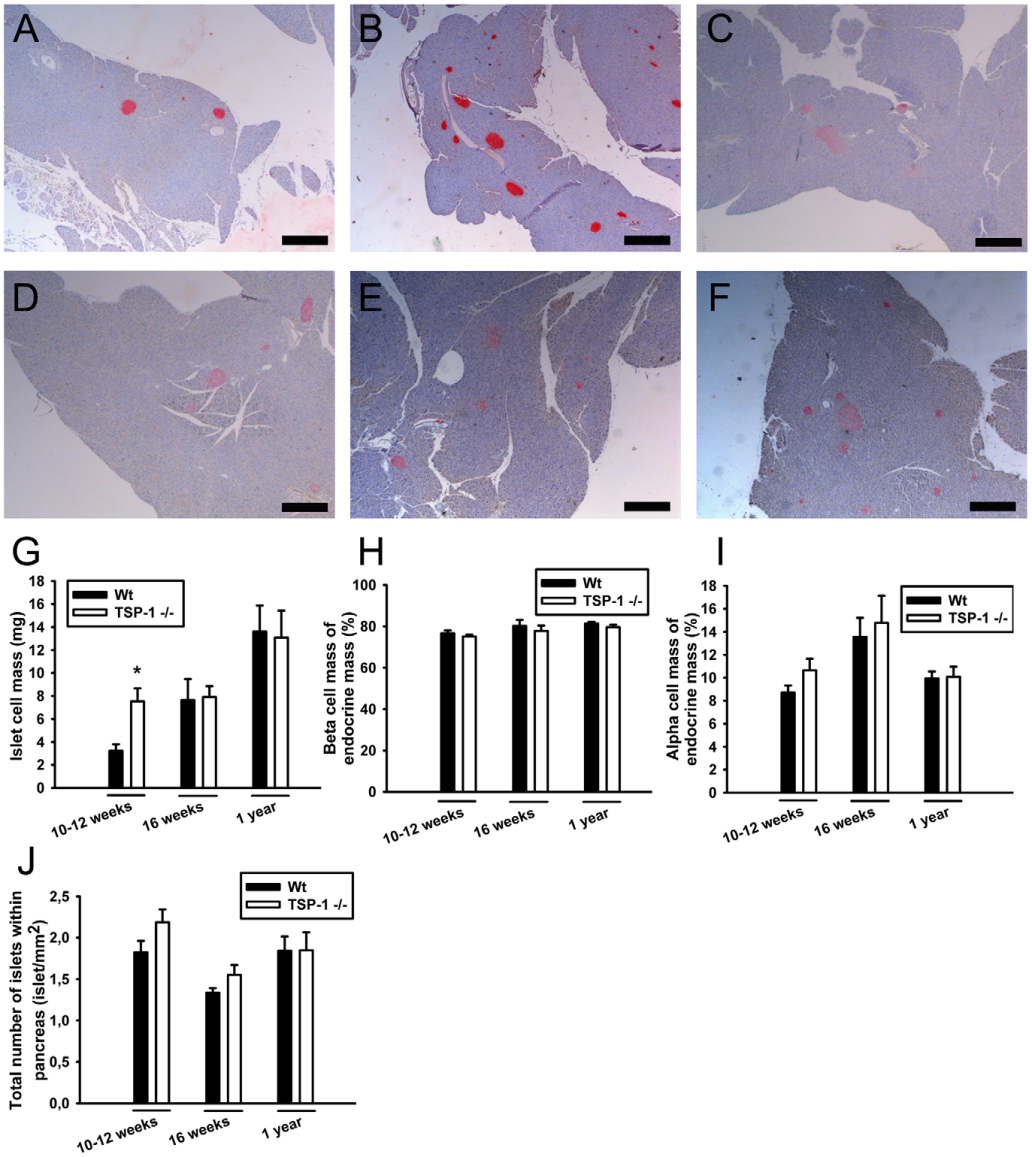
Age-dependent changes in islet and beta-cell mass of wild-type and thrombospondin-1 deficient mice. Micrographs of insulin-stained (red) pancreas from 10–12-week-old wild-type (A) and thrombospondin-1 deficient mice (B), 16-week-old wild-type (C) and thrombospondin-1 deficient mice (D), and one-year-old wild-type (E) and thrombospondin-1 deficient mice (F). Islet, beta- and alpha-cell mass in 10–12 week-old, 16-week-old and one-year-old wild-type and thrombospondin-1 deficient mice (G–I). Number of pancreatic islets in the same animals (J). All values are given as means±SEM for 5–8 animals in each group. * denotes P<0.05 when compared to age-matched wild-type animals. Scale bars in A–F are 400 µm.

The pancreas weights did not differ between wild-type and TSP-1 deficient mice at either 10–12 weeks, 16 weeks or one year of age (Data not shown). However, whereas the fraction of the pancreas occupied by islet cells and thereby the islet cell mass was higher in 10–12-week-old TSP-1 deficient mice when compared to age-matched wild-type mice, islet mass was similar in wild-type and TSP-1 deficient mice at 16 weeks or one year of age (Figure 1A–G). The fraction of the islets occupied by alpha-cells and beta-cells was similar in wild-type and TSP-1 deficient mice of all ages (Figure 1H–I). Therefore, both alpha- and beta-cell masses were increased in TSP-1 deficient mice when compared to wild-type mice at 10–12 weeks of age, but did no longer differ at 16 weeks or one year of age. The total number of islets per pancreas area was similar between wild-type and TSP-1 deficient mice of all ages (Figure 1J). Likewise, the different sizes of the islets were equally distributed between wild-type and TSP-1 deficient mice, respectively (Data not shown).

### Islet Vasculature

**Figure 2:**
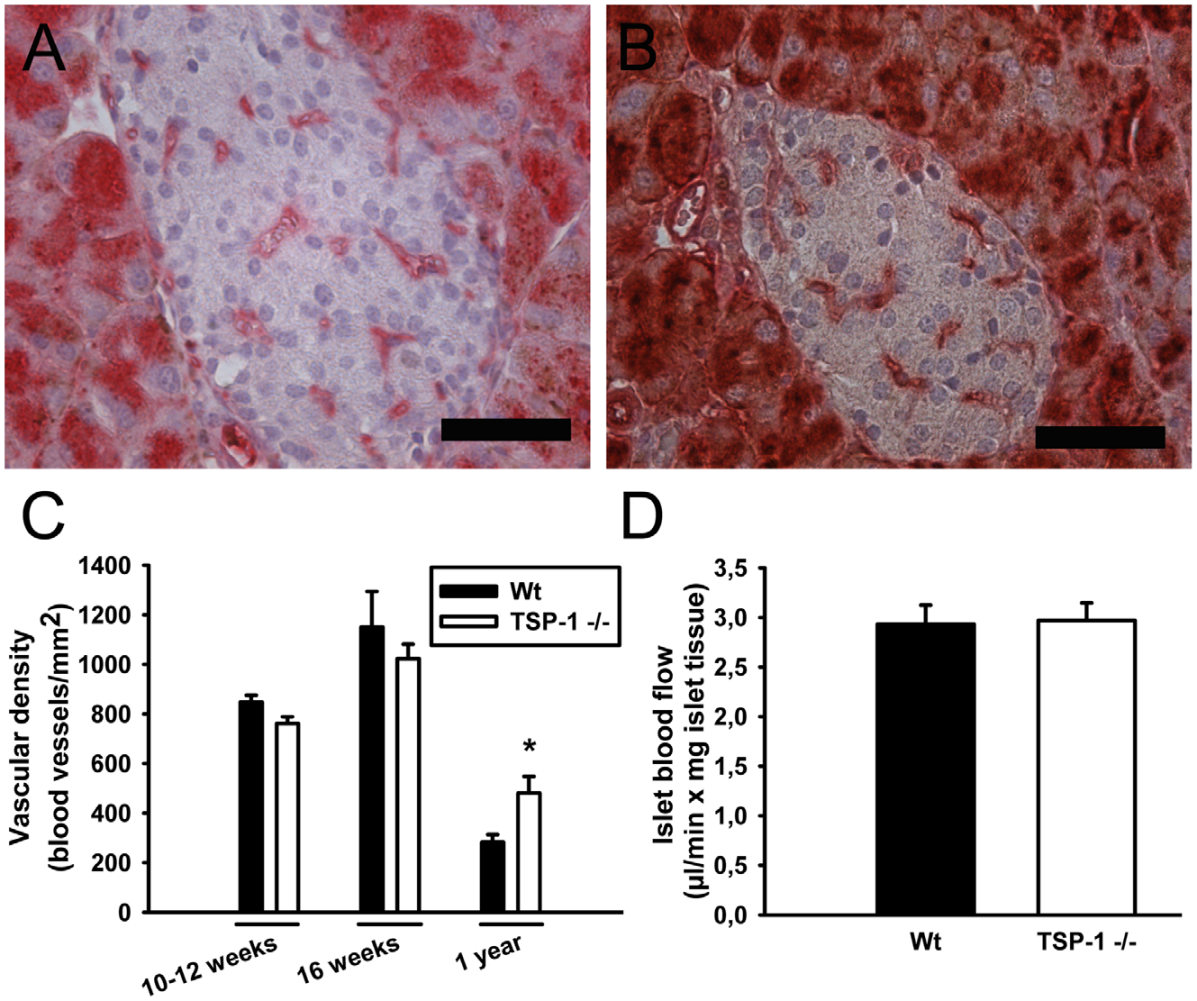
Islet vascularity in wild-type and thrombospondin-1 deficient animals. Micrographs of blood vessel-stained (Bandeiraea simplicifolia; red) pancreas form one-year-old wild-type and thrombospondin-1 deficient mice (A–B). Vascular density in 10–12 week-old, 16-week-old and one-year-old wild-type and thrombospondin-1 deficient mice (C). Islet blood flow in 10–12-week-old wild-type and thrombospondin-1 deficient mice (D). All values are means±SEM for 6–10 animals in each group. * denotes P<0.05 when compared to age-matched wild-type animals. Scale bars in A–B are 50 µm.

Pancreatic islets of wild-type and TSP-1 deficient mice were observed highly vascularized (Figure 2A–B). There were no differences in islet vascular density at 10–12 weeks or at 16 weeks of age between wild-type and TSP-1 deficient mice, whereas at one year of age TSP-1 deficient mice had more islet blood vessels than corresponding wild-type animals (Figure 2C). There was no difference in the blood perfusion of whole pancreata (Data not shown) or the pancreatic islets (Figure 2D) between 10–12-week-old wild-type and TSP-1 deficient islets.

### Glucose Tolerance and Islet Function

**Figure 3:**
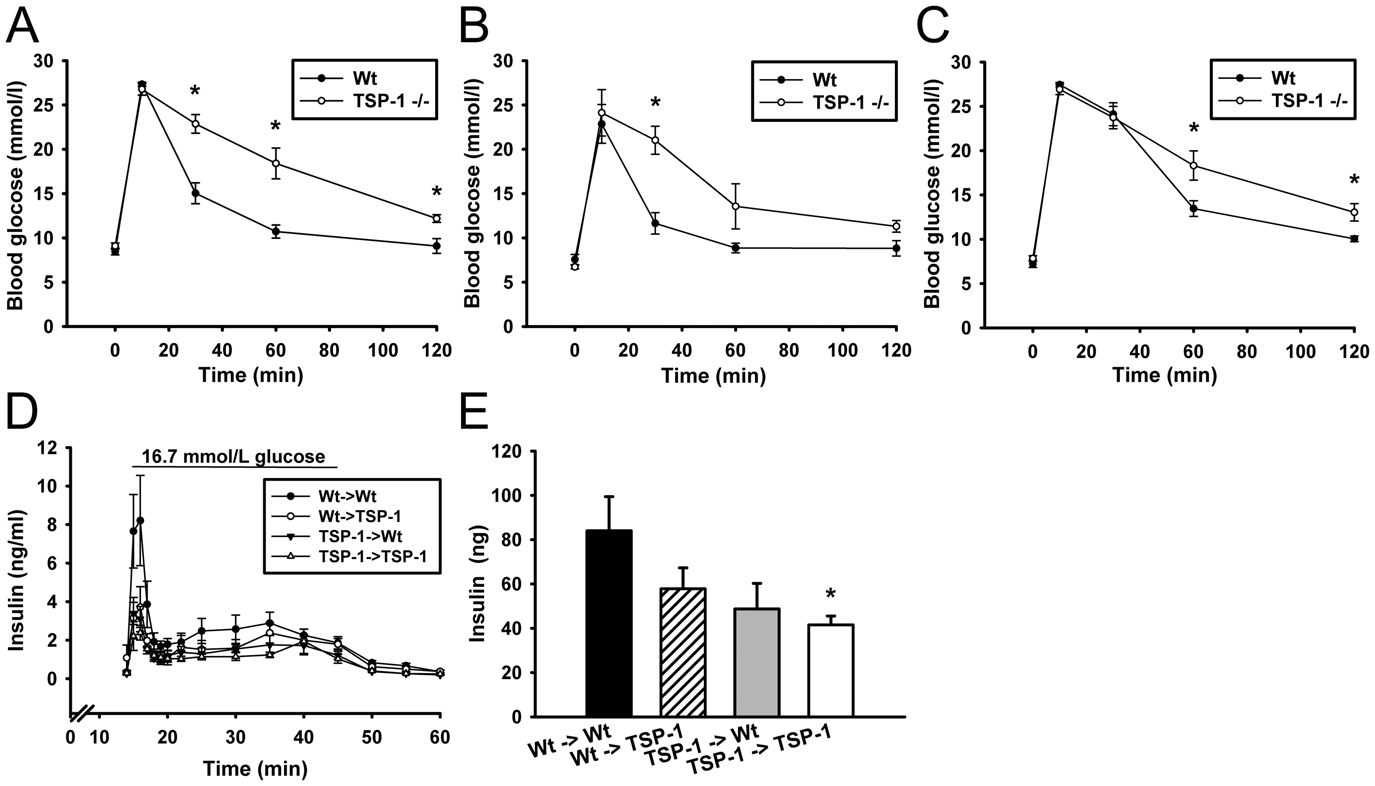
Beta-cell functional tests in wild-type and thrombospondin-1 deficient animals. Intravenous glucose tolerance tests (2.5 g/kg) in 10–12 week-old (A), 16-week-old (B) and one-year-old animals (C). Perfusion of islet grafts composed of wild-type or thrombospondin-1 deficient islets implanted to wild-type or thrombospondin-1 deficient animals (D) with calculations of total insulin release (area under the curve) for the high glucose period (15–45 min; E). All values are means±SEM for 5–14 animals in each group. * denotes P<0.05 when compared to age-matched wild-type animals.

Both wild-type and TSP-1 deficient 10–12-week-, 16-week and one-year-old mice showed normal non-fasting plasma glucose values (8.5±0.2 and 8.9±0.2 mmol/l for 10–12-week-old, 7.6±0.6 and 6.7±0.2 mmol/l for 16-week-old and 7.2±0.4 and 7.8±0.3 mmol/l for one-year-old wild-type and TSP-1 deficient mice, respectively). However, at all ages TSP-1 deficient mice showed impaired glucose tolerance after intravenous glucose administration (Figure 3A–C). Serum insulin levels 10 min after glucose administration were substantially lower in TSP-1 deficient mice when compared to wild-type mice at one year of age (24.6±6.8 vs. 3.8±0.9 ng/ml, P<0.05). Transplantation of adult TSP-1 deficient islets (animals 10–12 weeks of age) into wild-type recipients failed to reverse their beta-cell dysfunction. When challenged with high glucose (16.7 mmol/l) TSP-1 deficient islets implanted to wild-type mice responded as poorly as TSP-1 deficient islets implanted to TSP-1 deficient mice (Figure 3 D–E).

## Discussion

TSP-1 is an extracellular matrix bound glycoprotein and was the first naturally occurring protein inhibitor of angiogenesis to be reported [19]. It exerts its main anti-angiogenic effects by inducing apoptosis selectively in activated islet endothelial cells, i.e. those that are forming new blood vessels, but not quiescent endothelium [10]. It also inhibits angiogenesis by blocking the mobilization of pro-angiogenic factors, such as matrix metalloproteinase-9 and VEGF, and by inhibiting their access to co-receptors on the endothelial cell surface [20].

Young pups of TSP-1 deficient mice (<2.5 weeks of age) have been observed to be mainly morphologically normal, except for their pancreatic islets [11]. The islets of these mice displayed massive islet hyperplasia and hypervascularity. We have later showed the possibility to improve the revascularization of transplanted islets by use of TSP-1 deficient or TSP-1 siRNA treated islets for transplantation [18]. In the present study, we show that islet vascular changes in young TSP-1 deficient mice are transient and disappear before 10–12 weeks of age. At this latter age, vascular density and islet blood perfusion in TSP-1 deficient islets are similar to those in control islets. These results suggest that although early vascularization of islets postnatally or after transplantation is facilitated by low TSP-1 levels, the increased expansion of the endocrine cell mass in TSP-1 deficient animals during their first months, as reported in the present study, cause a normalization of the islet vascular density. Indeed, we have previously described similar findings of increased vascularity and blood perfusion in young obese-hyperglycemic (ob/ob) mice followed by normalization after the massive islet mass expansion that occur also in such animals [21]. An augmented vascular support has in other settings, e.g. during perinatal islet growth in normal rats and during rat pregnancy, been shown important to stimulate the islet endocrine cell proliferation [6], [22]. At one year of age the islets are for unknown reasons again hypervascular in TSP-1 deficient mice.

Lack of activation of TGFβ-1 has previously been shown to mediate the islet morphological and functional changes described in neonatal TSP-1 deficient mice [9], [11]. TGFβ-1 is well known to play an important role in pancreatic islet morphogenesis during rat pancreatic development by controlling the activity of matrix metalloproteinase 2 [23]. The present results imply that although TSP-1 and TGFβ-1 continue to exert effects on islet morphology postnatally, these effects are transient or compensated after the first months by other mechanisms. Interestingly, TGFβ has previously been described to block the mitogenic response of beta-cell to glucose [24], and overexpression of TGFβ-1 in transgenes has been shown to generate small islet cell clusters, but in increased numbers meaning that the overall islet cell mass does not become substantially diminished [25]. However, in TSP-1 deficient animals both islet numbers and islet size distribution were not significantly different from wild-type controls. Noteworthy, the morphological effects of TSP-1 and TGFβ-1 could be both direct and indirect. A possibility is that the observed early changes in islet and beta-cell mass are secondary to the alterations in beta-cell gene expression and function, e.g. induced by a dedifferentiated state or by sustained glucokinase activation postprandially (cf [26]).

We have previously characterized the function of young TSP-1 deficient mice, 10–12 weeks of age, and found that these animals are glucose intolerant due to defects in beta-cell function including defects in glucose-stimulated insulin release and (pro)insulin biosynthesis [9]. A more detailed investigation of isolated islets from TSP-1 deficient mice showed that they had an impaired capacity to oxidate glucose, which together with upregulation of uncoupling protein-2 (UCP-2) indicates a perturbed mitochondrial function. A further functional defect may be caused by the observed increased lactate dehydrogenase-A (LDH-A) levels, leading to augmented shunting of pyruvate into lactate, and decreased pancreatic and duodenal homeobox gene-1 (PDX-1) protein levels in the TSP-1 deficient islets. Interestingly, treatment of neonatal TSP-1 deficient mice with the TGFβ-1 activating sequence of TSP-1 showed that reconstitution of TGFβ-1-activation prevented not only development of islet morphological changes [11], but also beta-cell dysfunction in these mice. The present follow-up study shows that these functional defects in contrast to morphological changes are not transient, but persist at one year of age in these mice. At this latter age, both a glucose tolerance test and peak insulin values during this glucose load were similar to those at 10–12 weeks of age.

In additional experiments, we tried to reconstitute TSP-1 and thereby TGFβ-1 in TSP-1 deficient islets of adult mice by transplantation of islets into wild-type recipients. Islets cultured prior to transplantation have previously been shown to derive most, if not all, of their new vascular system from the recipient [27], [28]. However, in contrast to neonatal TSP-1 deficient islets that regained function after revascularization by TSP-1 positive blood vessels derived from the recipient [9], adult islets failed to recover. This indicates an irreversible beta-cell dysfunction induced by persistence of TSP-1 deficiency if not substituted early in life. The duration of TSP-1 deficiency is likely of importance for this, since induced TSP-1 deficiency by siRNA treatment caused only a transient beta-cell dysfunction parallel to the reversible decrease in TSP-1 expression for 1–2 weeks [18]. One reason for this may be glucose toxicity, but fed blood glucose levels were similar in TSP-1 and wild-type animals, and e.g endoplasmic reticulum stress is not seen in these animals [9]. Moreover, TSP-1 deficient animals were on C57BL/6 background, which is resistant to islet dysfunction induced by high glucose levels [29]. A possibility may instead be in line with the notion that a dedifferentiated state of beta-cells is not easily fully reversible [30], in this case by reconstitution of TSP-1.

We conclude that TSP-1 deficiency in islets causes changing vascular and endocrine morphological alterations postnatally, but is coupled to a chronic beta-cell dysfunction. Thus, paracrine support of the islet endothelial product TSP-1 is identified as an important factor for maintenance of glucose-stimulated insulin secretion and thereby glucose homeostasis.
